# Genome Sequence of a Persistent Campylobacter jejuni Strain, 2016-IZSVE-19-111250

**DOI:** 10.1128/MRA.01033-20

**Published:** 2020-10-08

**Authors:** Arianna Peruzzo, Barbara Salerno, Alessia Tiengo, Sara Petrin, Lisa Barco, Carmen Losasso, Massimiliano Orsini

**Affiliations:** a Laboratory of Microbial Ecology and Genomics of Microorganisms, Italian Health Authority and Research Organization for Animal Health and Food Safety, Legnaro, Italy; b Reference Laboratory for Salmonellosis, Italian Health Authority and Research Organization for Animal Health and Food Safety, Legnaro, Italy; University of Rochester School of Medicine and Dentistry

## Abstract

In this report, we present the whole-genome sequence of a Campylobacter jejuni strain isolated recursively for the last 3 years from an Italian poultry farm.

## ANNOUNCEMENT

Campylobacter jejuni is a Gram-negative bacterium causing campylobacteriosis (1), which is the most commonly reported zoonosis in humans in the European Union since 2005 (2).

Interestingly, by monitoring the presence of C. jejuni in an Italian poultry farm for 3 years, across time and in different conditions (pre- and postdisinfection), we recursively isolated a C. jejuni strain displaying sequence type 50 (ST 50). In this work, we characterized the isolate collected in the last postdisinfection sampling (February 2019).

C. jejuni was isolated in a poultry farm located in Lozzo Atestino, Padua, northeast Italy, from fecal sponge following official protocol ISO 10272-1:2017 (3), which is based on bacterial growth on selective Preston medium (4), under microaerophilic conditions at 41.5 ± 1°C for 48 h. After biochemical and morphology tests, which confirmed the isolate as a *Campylobacter* sp., one colony was submitted for DNA extraction using a QIAmp DNA minikit (Qiagen). Species confirmation was made by PCR using the gene *mapA* as the target (5). The genomic DNA was quantified and diluted to prepare libraries for sequencing using the Nextera XT DNA library prep kit (Illumina). Libraries were checked for concentration and quality using Qubit and 2200 TapeStation (Agilent) instruments, respectively. Deep sequencing was performed on the Illumina NextSeq 550 platform using the NextSeq 500/550 high-output cartridge version 2 (300 cycles, 2 × 150-bp paired-end reads).

The sequencing returned 3,775,114 read pairs, corresponding to a theoretical coverage of about 350×. Quality control, trimming, and preliminary genome assembly were carried out with the Orione platform (6) using “Fastq quality and positional trimming” and SPAdes version 3.13 (7) with default parameters, which returned 7 contigs (average length, 237,813 bp; *N*_50_, 659,440 bp). Default parameters were used for all software unless otherwise specified. The closest reference was chosen by submitting contigs to KmerFinder version 3.0.2 (8) (https://cge.cbs.dtu.dk/services/KmerFinder/), which returned the C. jejuni strain WP2202 (GenBank accession no. NZ_CP014742.1) genome, used only as a guide for scaffolding using ABACAS software version 1.3 (9). The scaffolding process returned a pseudomolecule containing virtual gaps as stretches of Ns. These virtual gaps were filled by alternating rounds of GapFiller version 2.1.1 (10) followed by Pilon version 1.23 (11) to improve the gap-filling process and sequence quality. Once this was completed, the pseudomolecule was rotated to start from the *dnaA* gene (identified by a preliminary annotation performed using Prokka and not further considered). This step produced a tail-to-end gap which was filled as described above.

The final assembly, consisting of only one circular contig (total sequence length, 1,685,647 bp; GC content, 30.5%), was submitted to GenBank and annotated by the NCBI staff using PGAP version 4.12 (https://www.ncbi.nlm.nih.gov/genomes/static/Pipeline.html) (12). Annotation returned 1,750 genes, 1,645 coding sequences, 9 full rRNA operons, 53 pseudogenes, 39 frame-shifted genes, 1 CRISPR array, 3 noncoding RNAs (ncRNAs), and 40 tRNAs.

Among the annotated genes, some encode multidrug efflux system transporters (such as *cmeABC*), organic solvent tolerance protein (*ostA*), various metal resistance proteins (*copA*), and heavy metal transport/detoxification proteins. The presence of these genes could contribute to the resistance to treatments of this strain, then defining its peculiar persistence (13–16).

### Data availability.

The complete genome has been deposited in the NCBI assembly database with accession no. CP053659.1. The raw reads were uploaded to the European Nucleotide Archive database under the accession no. ERS4545797.
